# Fluid Consumption by Mexican Women during Pregnancy and First Semester of Lactation

**DOI:** 10.1155/2014/603282

**Published:** 2014-02-02

**Authors:** Homero Martinez

**Affiliations:** ^1^RAND Corporation, 1776 Main Street, Santa Monica, CA 90406, USA; ^2^Hospital Infantil de México “Dr. Federico Gómez”, Dirección de Investigación Médica, Dr. Márquez No. 162, Col. Doctores, Delegación Cuauhtémoc, 06725 Mexico city, DF, Mexico

## Abstract

The objective of this study was to describe daily fluid consumption in a sample of pregnant or lactating adult women. Women between 18 and 45 years of age, residents of Mexico City, stratified by socioeconomic status were asked to register their total fluid intake during 7 consecutive days. A total of 153 pregnant and 155 lactating women were recruited. On average, they drank 2.62 L/day and 2.75 L/day, respectively. Forty-one percent of pregnant women drank less than the recommended 2.3 L fluids/day, and 54% of women drank less than the recommended intake of 2.7 L/day during the first semester of lactation. Plain water contributed to 33% of total fluid intake, and sugar-sweetened beverages (SSB) contributed to 38% of total fluid intake. Up to 50% of pregnant and lactating women drank more than 1 L/day of SSB, which contributed to 632 kcal/day (27.5% of recommended dietary intake) and to 700 kcal/day (28% of recommended dietary intake), respectively. The high rates of overweight and obesity found in Mexican population, particularly among women, should alert us to the consumption of SSB during pregnancy and lactation, as excessive intake of these beverages may increase the risks of obesity, diabetes mellitus, and other chronic disorders.

## 1. Introduction

Water is the most common element in the human body, accounting for about 60% of total body weight [1]. More than half of the water present in the human body (~60–70%) is found inside the cells; the rest is distributed between the circulatory system (including blood and lymph) and interstitial tissue. Most of the water in the body comes from ingestion, either as beverages or food; only a small fraction comes from cell metabolism. In usual conditions, daily beverage intake varies between 2.5 and 3.0 liters [2]. However, recommendations about daily fluid intake vary widely between countries [3, 4].

In Mexico, the Ministry of Health convened a panel of experts to review current scientific evidence and make recommendations about fluid and food intake [5]. Based on their review and expert advice, the Ministry of Health recommended that consumption of water and beverages with a very low energy content or no added calories, including skim milk, is preferable to those of higher energy content. Specifically, this committee recommended consumption of 750–2000 mL (6–8 glasses) of water per day.

For other beverages, the recommendation included daily consumption of 0–500 mL of partially (1%) or completely skimmed milk or soy-based milk without sugar; 0–1000 mL of unsweetened coffee or tea; 0–500 mL of noncaloric beverages, which may include noncaloric artificial sweeteners; or 0–125 mL of fruit juices. Consumption of whole milk, sweetened beverages with low nutrient content (like bottled soft drinks, juices, home-prepared fruit drinks, or sweetened coffee/tea) is discouraged [5]. This committee did not make specific recommendations for women, but other authors have recommended that fluid intake should increase over an average of 2000 mL by 300 mL/day during pregnancy and 750 mL during lactation [6].

Previous information about fluid consumption in the Mexican population, collected by the national nutrition survey of 2006, shows that adults drink, on average, 1721 mL/day, of which 889 mL corresponds to water [5, 7]. However, there is no information about fluid consumption by women during pregnancy, or lactation. To fill in this gap, the objective of the present study was to describe the amount and type of fluid consumed by a sample of adult, pregnant, or lactating Mexican women living in the urban area of Mexico City.

## 2. Materials and Methods

A cross-sectional, observational study was carried out between February and April 2011. The sampling frame was taken from the basic geostatistical areas defined by the Mexican National Institute of Geography. Households were selected at random, and interviewers asked if there was a pregnant or lactating woman between 18 and 45 years of age who met the inclusion criteria. If so, she was invited to take part in the study. Sample size was calculated resorting to a formula to calculate a mean on a very large population: *n* = (*Zα*∗*S*)2/*d*2, where *n* is the number of subjects in the sample to be selected from the population, *Z* is the value associated with the desired confidence level, *S* is the estimated standard deviation in the population (based on experience or on a previous study), and *d* is the maximum measurement error allowed by the researcher [8]. For our specific calculation, the value of *S* was taken from the values of daily consumption of beverages reported in a previous study that determined the consumption of water and other liquids in a Mexican population, using tools similar to those that apply in this study, which found a standard deviation of 718.8 mL relative to the average water consumption by women in the age of interest. The value of *Z* was set for a confidence level of 95% (*Zα* = 1.96), and the maximum allowable error set by the researcher (*d*) was 100 mL. A sample of 150 pregnant and 150 lactating women was used to determine fluid intake with a maximum allowable measurement error of 115 mL.

Inclusion criteria for pregnant women included physiological pregnancy, diagnosed by two or more of the following criteria: absence of regular menstrual period for two or more consecutive months, abdominal growth compatible with pregnancy, positive urine test, and ultrasound. Classification of trimester of pregnancy was done on the basis of date of last menses, as other methods, like ultrasound, were not universally available for the study population. Inclusion criteria for lactating women included being in the first semester of lactation, regardless of whether this was exclusive or complemented by formula feed. In addition, women needed to be able to understand how to fill the fluid consumption diary used to collect data, as demonstrated during an initial training period.

Exclusion criteria included following a diet, either for weight reduction or for any medical condition, having type 2 or pregnancy-related diabetes mellitus, or having any renal condition. Cases were excluded from the analysis if there was incomplete data recording, outliers (fluid consumption under 400 mL/day or over 6 L/day), or if the participant had an atypical week during data recording, such as taking a trip or engaging in unusual physical activity. Recruitment was done house-by-house, following stratified random sampling taking into consideration socioeconomic stratum, which was classified in 6 levels (A, B, C+, C, D+, D, from higher to lower) [9].

Data collection was carried out by asking participants to register all fluid intake during a 7-day consecutive period [10]. This method was based on a dairy previously used in other populations [11]. Participants were asked to write down what type of beverage they consumed, to identify which container was used, the amount consumed, and the time of day when consumption took place.

To facilitate data entry, the fluid consumption diary is profusely illustrated, including the most common types of containers (e.g., a glass, a cup, a bottle, etc.) to help identify the amount of liquid ingested. Based on previous experiences [11], participants underwent a preliminary training period, during which interviewers helped them fill in the diary according to simulated intake of different fluids.

Following the training period, each participant received the dairy, and the interviewer came back for a home visit on the fourth day of data collection to review data entry and to clarify any doubts that participants may have had. Throughout the data collection period, participants had access to a telephone line in case they had any question about the procedure. Interviewers came back home at the end of the 7th days of data collection to retrieve the diary.

The type of beverages included were plain water, tap (untreated, filtered, or boiled) or bottled (mineral, plain, or bubbly); dairy products, including cow's milk, *atole* (rice gruel), milk shake, ready-to-drink flavored milk, ready-to-drink shakes, powdered milk, and powder nutrient supplements; ready-to-drink yogurt; beverages containing lacto-bacilli; soy drinks; orangeade, fruit juices (natural or canned/bottled), fruit drinks, or fruit-flavored soft drinks; sugar-sweetened powders; unsweetened powders (i.e., to prepare iced tea); bottled/canned iced tea; bottled soft drinks (regular or light); coffee and tea (including instant coffee, tea in sachets, hot coffee/tea from dispenser, or coffee/tea bar); functional beverages (i.e., hydration fluids and energy boosters); and alcoholic beverages.

All participants gave their oral informed consent before data collection and received a copy of the consent form. Confidentiality was promised to every participant. There were no economic incentives for participating in this study.

Statistical comparisons were carried out by means of *t*-tests for continuous variables or chi-square for percentages. Comparison between three or more samples was done by analysis of variance (anova). Significant differences were reported with *P* ≤ 0.05 and ≤ 0.01. Statistical analyses were run with Quantum V.5.7 software.

## 3. Results

Three-hundred and sixteen adult women between 18 and 45 years old were invited to participate and 308 accepted and turned in complete data. Of them, 153 were pregnant and 155 were in the first semester of lactation. The distribution by age, physiologic status, trimester of pregnancy, and socioeconomic level is shown in Table 1.

**Figure 1:**
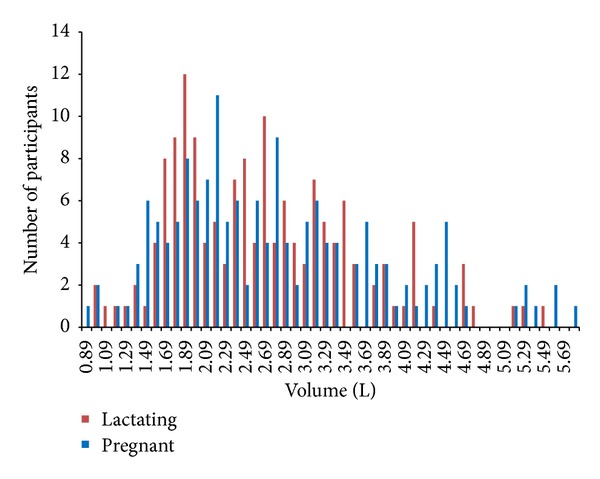
Volume of fluid intake by pregnant and lactating women.

The distribution of daily fluid consumption during pregnancy is shown in Figure 1. Mean fluid consumption was 2.62 L/day, with a bimodal distribution showing peaks at 1.89 and 2.69 L/day, skewed to the left. Forty-one percent of pregnant women drank less than the recommended 2.3 L/day. The distribution of daily fluid intake by women in their first semester of lactation is also shown in Figure 1. Mean consumption was 2.75 L/day, with a bimodal distribution showing peaks at 2.19 and 2.79 L/day, skewed to the left. Fifty-four percent of lactating women drank less than the recommended 2.7 L/day.

The average number of times that fluids were consumed throughout the day was 5.8, both during pregnancy and lactation. This average number of times showed very little variation across different ages and socioeconomic levels, ranging between 5.6 and 6.0 (data not shown).

Table 2 shows fluid intake according to the type of beverage consumed by pregnant and lactating women. It should be noted that both during pregnancy and lactation plain water contributed to 33% of total fluid intake, while SSB (including soft drinks) contributed to 38% of total fluid intake. Milk and dairy products contributed to 20% of total fluid intake during pregnancy and 18% of total fluid intake during the first semester of lactation. Comparing the volume of fluid consumption between pregnant and lactating women, there was a statistically significant difference in hot beverage consumption and soft drinks, both of which were consumed more during lactation.

**Figure 2:**
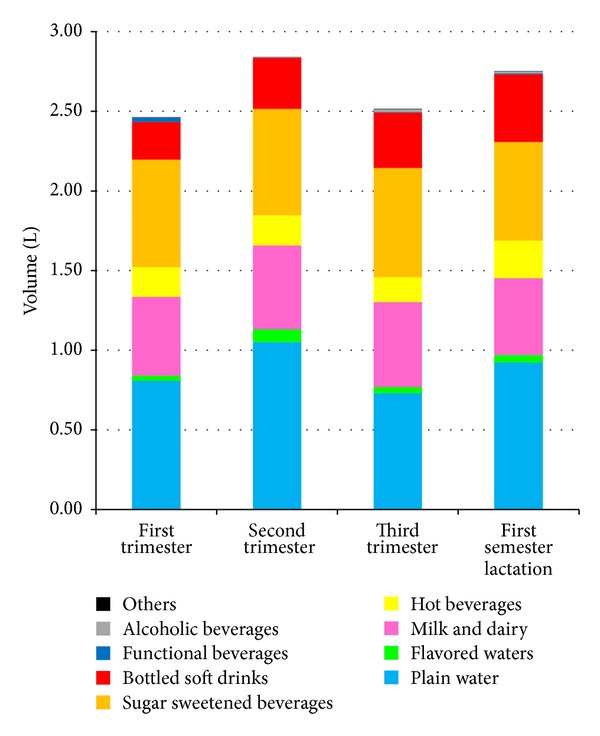
Fluid consumption by type of beverage, by trimester of pregnancy or semester of lactation.

Figure 2 shows fluid consumption according to the type of beverage, by trimester of pregnancy and first semester of lactation. Consumption of plain water increased slightly between the first (0.81 L) and the second (1.05 L) semester, decreasing by the third (0.73 L) (*P* < 0.05 by anova). Consumption of soft drinks followed a nonstatistically significant increasing trend (*P* = 0.08 by anova) as pregnancy advanced (9.9%, 11.6%, and 14.6%, resp. in the first, second, and third trimesters of pregnancy). The volume of SSB was relatively stable between these two time periods, while consumption of plain water showed a slight increase.

**Figure 3:**
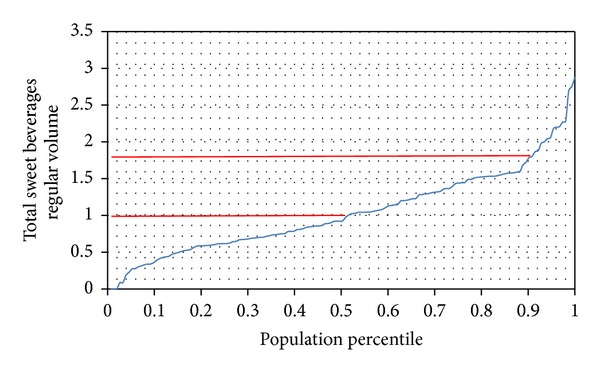
Percentile distribution of SSB intake by pregnant women.

Percentiles of consumption of SSB by pregnant women are shown in Figure 3. As may be seen, 50% of pregnant women drank more than 1 L of SSB/day, and the upper 10% consumed over 1.75 L/day. In 25% of pregnant women, SSB intake provided 632 kcal/day, equivalent to 27.5% of recommended daily energy intake for a pregnant woman [12]. In contrast, diet beverages represented 1% of the total intake of SSB, including soft drinks and home-prepared fruit water.

**Figure 4:**
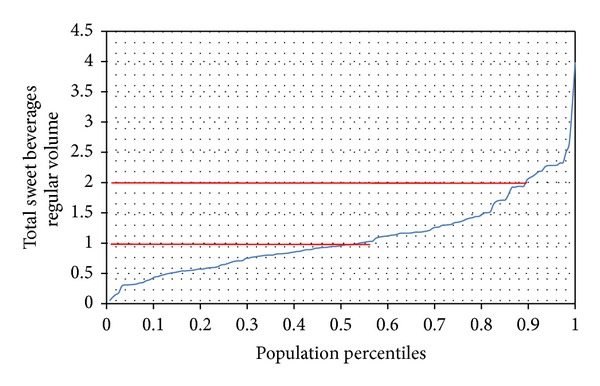
Percentile distribution of SSB intake by lactating women.

Figure 4 shows percentiles of intake of SSB during the first semester of lactation. As may be seen, during this period 50% of women drank more than 1 L of SSB, and 10% of lactating women drank over 2 L of SSB/day. Therefore, 25% of lactating women consumed 700 kcal/day, equivalent to 28% of the recommended energy intake during this period [12]. Diet beverages contributed 2% to total SSB.

## 4. Discussion

This is the first paper published that focuses on fluid consumption by a sample of women during the reproductive stage, including pregnancy and lactation. The main finding from this study was that 41% of pregnant women and 54% of lactating women consumed less than the recommended amount of fluids per day. A second finding was that consumption of SSB, including soft drinks, among this women accounted for 40% of fluid intake, which was greater than the consumption of plain water (33%), and showed an increasing trend as pregnancy progressed. These two findings may have important implications for health.

As for the first finding, it should be looked at by pointing that there is overall agreement within the scientific community about the need to consume water regularly and in enough amounts to maintain good health [2]; however, there is no general agreement about what should be the recommended amount of water intake. For example, the Institute of Medicine in the US recommends intake of 3.7 L/day for males and 2.7 L/day for females [4], while the European Food Safety Authority recently recommended intakes of 2.5 L/day for males and 2 L/day for females [3]. In Mexico, current recommendations for fluid intake have been set by an international panel of experts, based on available evidence [13], which supports intakes of 2.96 L/day for males and 2.16 L/day for females, which would amount to about 80% of daily fluid needs to be covered by beverages; a further 20% of fluid requirements should come from foods [5]. These recommendations recognize that a healthy diet should provide all energy and nutrient needs, with no need to complement their intake through beverages. In other words, plain water should suffice to provide for fluid requirements, and no additional beverages should be required to fulfill energy and nutrient requirements. However, individuals have specific preferences for specific flavors and drinks, leading this committee to include recommendations that encompass wide ranges for intake of different types of fluids [5]. It is worth mentioning that no specific recommendations were given for fluid requirements for pregnant or lactating women. This gap may be filled by looking at the recommendations of a different and independent group of researchers, who support the intake of 2.3 L/day and 2.75 L/day for pregnant and lactating women, respectively [12]. The importance of maintaining a good hydration status, particularly during periods of metabolic challenges and physiologic stress on the female organism like those present during pregnancy and lactation should not be overlooked.

The second finding may be a reason for concern in this particular population, as different studies have shown a statistically significant association between increased consumption of SSB and the risk of presenting overweight, obesity, metabolic syndrome, type-2 diabetes mellitus, and other chronic diseases associated with an increase in body fat [14–17]. Although several authors question the causal link between ingestion of SSB and the onset of obesity and related disorders, the World Health Organization has identified SSB as a “probable contributor” to the obesity epidemic [18]; and a recent systematic review focusing on the evidence between intake of SSB and weight gain has pointed out that there is sufficient evidence to support public health strategies that discourage consumption of SSB as part of the promotion of a healthy lifestyle [19]. The fact that we found a larger contribution from SSB to overall fluid intake than that provided by plain water should raise awareness about drinking practices among this population, particularly in the context of Mexico, where the rate of increase in prevalence of overweight and obesity among the adult female population is the highest in the world. Between 1988 and 2012, the amount of women 20–49 years old who were overweight increased from 25% to 35.3%, and obesity increased from 9.5% to 35.2% [20]. The most recent National Health and Nutrition Survey-2012 identified a prevalence of a medical diagnosis of type-2 diabetes mellitus of 9.2% among the adult population [20].

To properly interpret our findings it is important to recognize that there were several limitations in our design. Our study sample was not representative of the larger Mexican population. We carried out our study in Mexico City, the largest metropolitan area in the country, but although houses to be sampled were chosen randomly, we did not carry out a population-based random sample of all women of reproductive age. Likewise, we did not attempt to cover rural areas. Further, our design called for stratification of pregnant women by trimester of pregnancy, but accurate means of assessing pregnancy, like ultrasound, are not routinely available for all pregnant women. Clinical methods, including date of last menses, may under/overestimate age of pregnancy by as much as two weeks. As for lactating women, we focused on the first 6 months after birth of offering breast milk to their infant, irrespective of whether lactation was offered exclusively or supplemented by formula. Mother's thirst and hydration needs may be influenced by exclusive or partial breast-feeding [21, 22]. These caveats limit the external validity of our findings. Further, we did not attempt to evaluate the hydration status of the sample population. We did not collect any biological marker of hydration, like urine density, and we did not attempt to measure the contribution of water to overall fluid intake through ingestion of solid food. While the ambient temperature in Mexico City is fairly stable throughout the year and our study took place outside of the coldest or warmest seasons, ambient temperature and climate may have influenced fluid intake practices in our study population. Therefore, we cannot make any claim about the adequacy of the hydration status of participants. Lastly, our study design did not attempt to control for contextual or environmental factors, such as size of the family, availability of food outlets near home, or accessibility to specific types of beverages, all of which may influence fluid consumption practices.

In contrast, there were several robust aspects of the methods used that are worth highlighting. Estimation of nutrient intake is often carried out by applying quantitative records of food consumption, usually following a 24-hour recall of food intake. In these methods, fluid intake is often overlooked or underestimated, as emphasis is placed on the times of food consumption, but many persons drink fluids aside from the main meals or snack times. The use of 24-hour dietary recall methods underestimates fluid ingestion by as much as 500 mL/day when compared to a fluid-specific intake diary [23]. Therefore, accurate measurement of fluid intake is not easy to do outside of controlled conditions [24]. In our study, we resorted to the use of a fluid intake pictorial diary, which had been previously developed and used in Mexican population [11] and was focused on capturing all fluid intake over a consecutive 7-day period. All women participating in the study had a training period previous to actual data collection, and the field team was available to offer support or answer questions throughout the study, including a pre-, mid-, and end-of-study home visit. Our sample size was calculated based on previous information about the variability in fluid intake in an adult population, estimated by a similar method to the one actually used in this study [11]. Our stratified sampling design allowed us to collect information on a wide range of socioeconomic status as well as the three trimesters of pregnancy.

In summary, we believe that our study contributes new data to this largely underresearched topic. As the first study to document actual fluid consumption practices by pregnant and lactating women over the course of a consecutive 7-day period, the information presented here furthers our understanding of these practices and supports evidence-based health recommendations. Different health practitioners, including obstetricians, nurses, midwives, and general practitioners, may find these data useful to promote recommendations for a healthier life style [23]. The contribution of our study to the epidemiological evidence related to food and fluid intake, particularly in settings such as Mexico where the rising prevalence of overweight and obesity has largely overcast that of under-nutrition, may support design of policies and programs focused on the promotion of better-informed eating and buying practices that support a healthy life-style.

## 5. Conclusions

This is the first study that documents fluid consumption by women during pregnancy and first semester of lactation. The main finding is that 41% of pregnant women and 54% of lactating women consumed less than the recommended fluid intake according to their physiologic status. A second finding is that SSB contributed a larger proportion to overall beverage intake than plain water. Given the importance of maintaining an adequate hydration status during pregnancy and lactation, this information may help health practitioners provide evidence-based recommendations about what type and amount of fluids to drink to promote healthy life styles.

## Figures and Tables

**Table 1:** Distribution by age, physiological status, trimester of pregnancy, and socioeconomic level of participant women.

Characteristic	Pregnant	Lactating	Total	Percentage
	(*n* = 153)	(*n* = 155)	(*n* = 308)	(%)
Age (years)				
18–24	30	48	78	25.3
25–29	41	34	75	24.4
30–34	50	29	70	25.6
35–39	17	34	51	16.6
40–45	15	10	25	8.1
Trimester of pregnancy				
First	42	—	42	27.5
Second	56	—	56	36.6
Third	55	—	55	35.9
Socioeconomic level*				
A/B/C+	34	21	55	18
C	64	68	132	43
D+/D	55	66	121	39

*The highest s-e level corresponds to “A” [9].

**Table 2:** Daily fluid consumption by pregnant and lactating women, according to the type of beverage consumed.

	Pregnant *n* = 153		Lactating *n* = 155	
	Volume (L)	Percentage (%)	Volume (L)	Percentage (%)
Total	**2.6**	**100**	**2.8**	**100**
Water	0.9	33.2	0.9	33.6
Flavored water	0.05	1.9	0.05	1.7
Milk and dairy	0.52	19.9*	0.5	17.5*
Hot beverages	0.18**	6.8**	0.2**	8.5**
Sugar sweetened beverages	0.7	25.8*	0.6	22.6*
Soft drinks	0.3**	11.7**	0.4**	15.4**
Functional beverages	0.01	0.4	0.01	0.2
Alcoholic beverages	0.01	0.3	0.01	0.47
Other beverages	0.00	0.05	0.00	0.07

*P < 0.05.

**P ≤ 0.01.
